# Holding on to the indispensable medication –A grounded theory on medication use from the perspective of persons with medication overuse headache

**DOI:** 10.1186/1129-2377-14-43

**Published:** 2013-05-22

**Authors:** Pernilla Jonsson, Annika Jakobsson, Gunnel Hensing, Mattias Linde, Crystal Dea Moore, Tove Hedenrud

**Affiliations:** 1 Institute of Medicine, Sahlgrenska Academy, University of Gothenburg, PO Box 453SE 405 30, Gothenburg, Sweden; 2 University of Gothenburg Centre for Person-Centred Care (GPCC), Sahlgrenska Academy, University of Gothenburg, Gothenburg, Sweden; 3 Department of Neuroscience, Norwegian University of Science and Technology, Trondheim, Norway; 4 Norwegian National Headache Centre, St. Olav’s University Hospital, Trondheim, Norway; 5 Institute of Neuroscience and Physiology, Sahlgrenska Academy, University of Gothenburg, Gothenburg, Sweden; 6 Department of Social Work, Skidmore College, Saratoga Springs, USA

**Keywords:** Headache, Medication use, Medication overuse headache, Qualitative study, Grounded theory, Patient perspective

## Abstract

**Background:**

Medication overuse headache (MOH) is a chronic headache disorder, caused by overuse of acute medication. To date, it remains unclear why some people overuse these medications. The aim of this qualitative study was to explore how individuals with MOH use medications and other strategies to manage headaches in their daily lives, and their thoughts about their own use of acute medication. Our intention was to develop a theoretical model about the development of MOH, from the perspective of those with MOH.

**Methods:**

Data collection and analysis were conducted according to grounded theory methodology. The participants were recruited via newspaper advertisements. Fourteen persons with MOH were interviewed in individual qualitative interviews.

**Results:**

The basic process leading to medication overuse was *holding on to the indispensable medication*. The acute medication was indispensable to the participants because they perceived it as the only thing that could prevent headaches from ruining their lives. The participants perceived headaches as something that threatened to ruin their lives. As a result, they went to great lengths trying to find ways to manage it. They tried numerous strategies. However, the only strategy actually perceived as effective was the use of acute medication and they eventually became resigned to the idea that it was the only effective aid. The acute medication thus became indispensable. Their general intention was to use as little medication as possible but they found themselves compelled to medicate frequently to cope with their headaches. They did not like to think about their medication use and sometimes avoided keeping track of the amount used.

**Conclusions:**

This qualitative study adds understanding to the process via which MOH develops from the perspective of those having MOH. Such knowledge may help bridge the gap between the perspectives of patients and health-care professionals.

## Background

Although medication use is the prevailing method for treating pain, excessive use of acute medication is usually not a successful strategy, particularly in the case of headaches where it may lead to medication-overuse headache (MOH), a chronic headache disorder with daily or near-daily symptoms, caused by overuse of acute headache medication [1,2]. This may, in turn, lead to negative consequences such as a higher disease burden [3], reduced quality of life [4,5] and potentially harmful physiological effects [6,7].

To date, it remains unclear why some persons with headaches overuse acute medications [6,8]. Improved understanding of the decision-making process concerning medication use among those who develop MOH seems to be a key issue. Several studies have described this process in persons with other types of headache [9-11]. These studies suggest that they weigh the possible pros and cons of medication use and other factors before deciding whether to medicate. This is in line with Horne and Weinmans’ model for beliefs about medicines, in the context of chronic illness, which hypothesizes that patients engage in an implicit risk-benefit analysis, in which beliefs about the necessity of their medication are weighed against concerns about the potential adverse effects [12]. Several studies have found that these beliefs are related to medication behaviour [13-18]. However, such risk-benefit analyses do not account for the fact that persons with MOH overuse acute medication despite negative consequences such as increased headache frequency. In the only study identified concerning decision-making in MOH, Lauwerier et al. [8] used a functional coping perspective and found that patients who primarily focused on the problem of pain as one that had to be solved were at a higher risk of developing MOH than those who tried to disengage from the problem and focus on other areas of life in stead.

Another way of regarding the overuse of acute medication in MOH is to focus on aspects of addiction and dependence. Some of the medications used in MOH (e.g., opioids) are indeed addictive, but there is no evidence for addiction to triptans or to simple analgesics [6]. There is an on-going discussion as to whether MOH should be considered an addictive disorder or not [19-23]. Some studies indicate that many individuals with MOH do fulfil criteria for addiction, whereas others have not found any difference concerning addiction between persons with MOH, migraineurs, and the general population [19,21,22,24].

Few studies concerning medication use are based on headache sufferers’ own statements [10,25-27], and none has been identified concerning MOH. In other disorders, qualitative studies have shown that patients use medications to retain their function [28], e.g. the overall expectancy among patients with rheumatoid arthritis was that the medication would minimize the personal impact of the disorder [29], and asthma patients described using medications in order to be able to live normally [28]. Qualitative research is thus valuable when it comes to exploring research questions such as illness behaviour and patients’ choices [30,31]. Knowledge about the thoughts of persons who develop MOH may promote the development of new strategies for prevention and care. This is important since MOH is a considerable public health problem, with negative implications for patients’ everyday lives [3,5], as well as for society [32].

Against this background, the aim of this qualitative study was to explore how individuals with MOH use medications and other strategies to manage headaches in their daily lives, and their thoughts about their own use of acute medication. Our intention was to develop a theoretical model about the development of MOH, from the perspective of those with MOH.

## Methods

### Design and method description

Grounded theory was chosen since it is a qualitative research method that is well suited for studying how people manage problematic situations in their lives [33,34]. It offers a systematic procedure for generating theories that are grounded in empirical data and describe how people’s constructions of reality are manifested in behaviours [33]. The method is thus suitable for the study of how thoughts about headache and medication use can lead to overuse and the development of MOH.

By interviewing people with MOH, we attempted to explore behavioural patterns underlying the development of MOH. We chose this approach to allow the participants to describe their thoughts and actions in their own words [35]. An essential feature of grounded theory research is the continuous cycle of collecting and analysing data [33]. Thus, we started the analysis as soon as the first set of data was collected and the subsequent data collection was guided by the research question and the developing theory.

### Data collection

Participants were recruited through advertisements in the national journal of a headache patient organisation (once, September 2010) and in a local daily newspaper (twice, October 2011 and January 2012). Inclusion criteria were age ≥18 years, ability to speak Swedish and a diagnosis of MOH, according to the 2006 International Headache Society appendix criteria [1].

In total, 39 eligible participants expressed interest in participating. This allowed us to make a purposive selection to obtain as much variation as possible with regard to age, sex, employment status and headache history. Data were collected through 14 individual qualitative interviews, Table 1. The average interview lasted 58 minutes (range 25–113 minutes). The participants mean age was 58 years (range 36–64 years) and they had various occupations, e.g. teacher, assistant nurse, plumber, psychologist, secretary and economist. One was unemployed and two were on disability pension due to headache. The others worked full-time or part-time.

**Table 1:** Characteristics of the participants

	**N**	**%**
Total	14	100
Age (years):		
30-39	2	14
40-49	4	29
50-59	5	36
≥60	3	21
Sex		
Female	9	64
Male	5	36
University education		
Yes	8	57
No	6	43
Living with partner		
Yes	11	79
No	3	21
Having children		
Yes	12	86
No	2	14
Self-reported migraine		
Yes	10	71
No	4	29

All participants reported having daily or near daily headaches. Ten participants had self-reported migraine, mostly in combination with tension type headache, and four reported only tension type headache. Nine participants overused triptans, usually in combination with analgesics such as acetylsalicylic acid, ibuprofen or paracetamol. The other five participants were only using analgesics. One participant was overusing an opioid-containing analgesic.

All interviews were conducted in Swedish by the first author (PJ). The quotes presented in the article have been translated. The participants were first asked the opening question “Could you tell me about your headaches?” They were then asked questions about their headaches and daily life, strategies to manage headaches, use of medication and thoughts about using less medication. They were encouraged to tell their stories freely and probing questions were used to obtain as much detail as possible. All interviews were held at the University of Gothenburg, with the exception of one, which took place at the participant’s work place (a hospital). Each interview was audio-recorded and transcribed verbatim.

The first author (PJ) made a preliminary MOH diagnosis before a potential participant was included. After the interview, all participants talked on the phone to a neurologist, specialising in headache (ML), for verification of the diagnosis. This was also an opportunity for the participant to ask questions. Although it would have been convenient to have the diagnosis confirmed before the interview, we chose this procedure to avoid the risk that the consultation would affect the interview. In total, 15 interviews were conducted but one had to be excluded because the participant did not meet the criteria for MOH.

The researchers involved in the study have different professional backgrounds. Two of them are pharmacists (PJ and TH), two have a background in social work (GH and CDM), one is a nurse (AJ) and one is a physician (ML). Only ML has clinical experience of working with headache patients. TH, ML and PJ have previous experience of headache research and AJ, GH and CDM are experienced in using qualitative research methods.

The regional ethical review board in Gothenburg approved the study (Reference No. 293-10/2010). The participants received verbal and written information that participation was voluntary, that they could withdraw without further explanation and that confidentiality was guaranteed. Written informed consent was obtained.

### Analysis

The open coding began as soon as the first interview was completed, through reading the text line by line and creating codes. The main author (PJ) conducted the open coding and two co-authors (TH and AJ) read each interview. All three discussed the coding and analysis in meetings between each subsequent interview. The analysis proceeded until consensus was reached. Throughout the analysis, constant comparison and asking questions about the data were important tools. Constant comparison involves comparing each situation with other situations for similarities and differences [33], and useful questions includes: “What is going on?” and “What is expressed here?”[35].

The coding process moved on to the next level of analysis, in which the codes were clustered into categories. The next step, axial coding, included exploration of the connections between categories and subcategories to develop conceptual density. In this process, three main categories and several subcategories were defined. The core category developed in the theoretical coding process. In this step, we analysed the data with the aim of finding pieces of data that would help integrate and refine the categories in order to obtain a dense, saturated theory [33]. The theory developed when the core category was linked with the three main categories.

After 11 interviews, the preliminary categories and the emerging theory were discussed by all co-authors and at a seminar with researchers from different disciplines/professions. After 14 interviews and analyses, no more meaningful information was gained, indicating theoretical saturation. During the analysis, ideas and preliminary theoretical reflections were written down in memos to help with the generation of a theoretical model [33].

## Results

**Figure 1:**
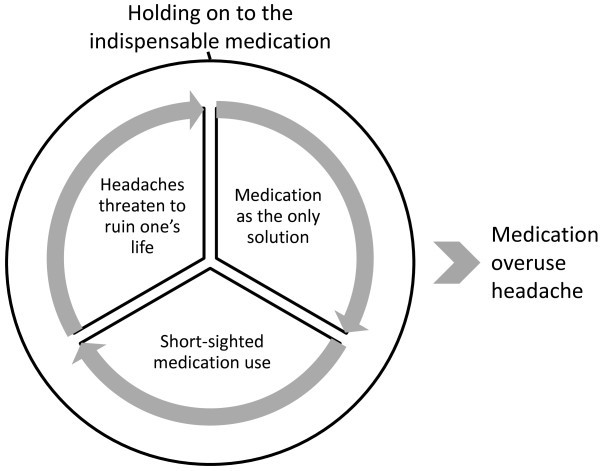
**The basic process leading to medication overuse headache.** The process of holding on to the indispensable medication, which eventually leads to MOH, includes three parts: headaches threaten to ruin one‘s life, medication as the only solution, and short-sighted medication use.

The data revealed three main categories: *headaches threaten to ruin one‘s life, medication as the only solution* and *short-sighted medication use*. The main categories and their subcategories are presented in Table 2. The core category, labelled *holding on to the indispensable medication,* was central to the data and pulled all three main categories together, as illustrated in Figure 1.

**Table 2:** Subcategories of main categories

**Main categories**	**Subcategories**
	
Headaches threaten to ruin one’s life	Headaches are unbearable
	An extra burden in everyday life
	Having to make life adjustments
	Struggling to be able to work
	Being forced to cancel important events
	
Medication as the only solution	Searching for explanations
	Testing numerous strategies
	Scepticism towards prophylactic medication
	Resignation: Nothing but the medication helps
	Always having the medication at hand
	
Short-sighted medication use	Taking medication because one has to, not because one chooses to
	Focusing solely on the headaches when deciding whether to medicate
	Avoidance of tracking medication use
	Increased medication use during stressful periods in life
	Perceptions about the link between increasing headaches and medication use

### Core category: Holding on to the indispensable medication

The basic process leading to medication overuse was *holding on to the indispensable medication,* Figure 1. The participants viewed their acute medication as indispensable, because they perceived it to be the only thing that was effective against their headaches. They believed that without the medication, the negative consequences of headaches would ruin their lives. In that sense, they depended on the medication to maintain their current lifestyle.

The participants’ perceived headaches as something that threatened to ruin their lives (*headaches threaten to ruin one‘s life)* and despite extensive efforts, they had been unable find any other effective aid besides acute medication. They thus regarded the medication as the only effective aid (*medication as the only solution)* and as a result, the medication had become indispensable. They avoided questioning their medication use by focusing on the headaches rather than keeping track of the amount of medication used (*short-sighted medication use*). One participant concluded:

"These triptans are the only thing I have found that really helps, so that I can live my life and do what I want to in the daytime, even during the bad days. So… if it stops, or if I am not allowed to take it anymore, because I have taken too much /…/ Just thinking about it makes me very nervous. Because my own assessment of the situation is pretty much that I would have to go on disability pension then. (No. 4)"

### Main categories

The three main categories and their subcategories are described below. For an overview, see Table 2.

### Headaches threaten to ruin one’s life

Headaches affected important areas of the participants’ lives in ways that made life feel less worth living. This was partly because the disorder itself was unbearable, and partly because of its consequences for other parts of their lives. The headaches were an extra burden in their everyday lives. Because of the headache, they had to make life adjustments and were unable to live their lives the way they wanted to. They struggled to keep working. The headaches were unpredictable, which meant that they often had to cancel things they had planned.

#### Headaches are unbearable

The participants described being beside themselves with pain during migraine attacks. They described laying down in a room that was dark, quiet and cool and waiting for it to pass.

"It is a terrible thing. I can’t do anything. No one can talk to me then… There are, well, there are suicidal thoughts. It is so awful. Then I lie down in a room that is cool… with a bucket next to me. (No. 7)"

Some were afraid of the pain, afraid of the next attack. Those who had tension type headache as the primary headache described the pain as disturbing rather than frightening.

#### An extra burden in everyday-life

The headaches were an extra burden in addition to the regular daily tasks such as going to work, taking care of children and domestic work. They also made it difficult to concentrate, think clearly and make good decisions.

"When I should be making decisions and thinking clearly, then I don’t and my head keeps aching, you know, and it takes the focus away from what you are supposed to be thinking about. (No. 13)"

Because of the extra burden, they could only manage the most important parts of life, usually their children and work, and they had to refrain from other things. They were sad that they had to forego things like a social life, exercise, travel and hobbies.

#### Having to make life adjustments

Because of headaches, they had to make compromises and could not have the kind of life they would have liked to live. They had to make adjustments since they knew that they would have even more headaches if they pushed themselves too hard, whereas on the other hand, they did not want to let headaches take over their lives entirely by adjusting too much. Because of the required changes, life had become restricted.

"My life is very handicapped… or limited, if I could use that word… it is incredibly limited. Having these headaches controls my life, although I refuse to let it, it does in many ways. (No. 10)"

#### Struggling to be able to work

The participants struggled to go to work every day and to manage their tasks despite their headaches. They also thought that their headaches would affect more long-term factors such as wages and pensions and that they could eventually force them to choose a less demanding job or even early retirement.

"As for investing in me at work, concerning both salary and things like that, I think of that and… It should not have any influence, but I think it does anyway. (No. 2)"

They developed strategies to manage work despite headaches, such as working in the evening rather than the morning or planning projects so that there were extra time buffers in case the headaches became worse.

#### Being forced to cancel important events

As a result of their headaches, they constantly had to cancel important events at short notice. It was hard to disappoint others and be unable to participate. Sometimes they even avoided making appointments because they dreaded having to cancel them. Being unable to plan things was considered debilitating and limiting.

"I was handicapped in a way. I couldn’t decide that… I tried… life went on as usual, but I could not plan things. Then the headaches came the next day, so I just said, “no, I cannot come” e.g. out to meet friends for birthdays and such. It was really hard. (No. 6)"

Sometimes they sensed that other people were suspicious, presumably thinking that headaches were being used as an excuse. This suspicion made them angry and sad.

"Some people probably think that, “Oh my God, she has migraines all the time, is it because she is lazy and does not want to be here, or what?” Yes, it’s like an excuse to stay at home or something. (No. 9)"

### Medication as the only solution

The participants went to great lengths to find ways to manage their headaches. They searched for explanations, tested numerous treatments and made extensive lifestyle changes. They were sceptical towards prophylactic medication, but other than that, they had been willing to test a wide variety of strategies in the hope that they would improve their headaches. In their experience however, none of the efforts had led to significant improvement. The only thing that actually helped was the acute medication. Because of this, they eventually became resigned, accepting the acute medication as the only effective aid. The acute medication was thus perceived as indispensable, and they made sure that they always had it at hand.

#### Searching for explanations

They expressed a need to find out what was causing the headache and had tried to find a specific somatic explanation, such as neck problems, hormones, high blood pressure etc. At times, they worried that the headaches were a symptom of serious illness, such as a tumour or a stroke.

"I used to think that I probably have cancer in the head. (No. 8)"

The participants also considered the relationship between headaches and psychological factors such as stress, fatigue and depression.

"It becomes more stressful when I start working and then I get headaches too. That is how I think, anyway. (No. 12)"

#### Testing numerous strategies

They were searching for strategies to manage the headaches and tried almost anything that they believed might be effective, regardless of costs in terms of money or effort. Many had tried numerous strategies, including physiotherapy, yoga, massage, acupuncture, osteopathy, removing amalgam from their teeth, homeopathic remedies, chiropractic care, naprapathy, body awareness training, various naturopaths, diets etc.

"I have tried lots of treatments: have spent an incredible amount of money, during the last 10–13 years. (No. 10)"

They also made changes to their lifestyle, such as changing daily routines and avoiding trigger factors. An important issue was to avoid stress, e.g. by changing to a less demanding job, learning relaxation techniques and practicing the ability to say no. Many had been in psychotherapy or in specific headache schools. Sleep was also important. They were taking various measures to improve their sleep, e.g. going to sleep courses or taking sleeping pills.

#### Scepticism towards prophylactic medication

They were reluctant to use prophylactic medication because they did not want to medicate daily. They viewed the acute medication as necessary and because they already had to take so much of it, they were reluctant to add another medication (the prophylactic).

"If you keep medicating as much as I do all the time… with triptans too… then you could think that it is less harmful for the body to do only that, than to add yet another thing that you should take daily. (No. 4)"

They were also afraid of side effects. Some had experienced side effects and others had only read about them and become too frightened even to try the prophylactic medication.

"But then, when I read about the side effects, I got really scared, and I have not dared to try them [the prophylactic medication], so I never started using them. (No. 2)"

#### Resignation: Nothing but the medication helps

It was described as demanding to constantly be searching and testing new strategies and looking back, it became evident for some that none of the strategies had really improved the headaches. Some of the lifestyle changes had helped them live healthier lives in general, but the headaches had remained unchanged.

"…yoga and massage, and mindfulness and such things. It has kind of been important and has given me a lot. But it has not, as far as I can interpret myself… the migraine has… it continues with its frequency and intensity, much like it always has. (No. 4)"

The only thing that had really helped was the acute medication. They were disappointed that the other strategies had not provided any relief. For some, their resignation meant that they “treated themselves” to the medication whenever they felt that they needed it, without feeling guilty.

"I have, as I said, become kinder to myself that way; I kind of allow myself to take the medication when I feel that I need it, without feeling guilty. (No. 4)"

#### Always having the medication at hand

They carried the medication with them, wherever they went. Many carried it in a special box. Having the medication at hand made them feel calm and secure. They checked that they had put it in their bag or pocket before leaving home. If they realized that they had forgotten the medication, they became anxious and had feelings of panic, since they were afraid of having to endure an attack without medication.

"I have always got this little box with tablets in my pocket /…/ It is my security blanket. (No. 1)"

### Short-sighted medication use

Throughout the interviews, the participants described their use of medication as something they did because they had to, in order to manage their headaches, not because they chose to. They had a general intention to use as little medication as possible but found themselves compelled to medicate frequently to cope with the headache. They did not want to think about how much medication they used. Instead, they focused on the headaches. Decisions about when to medicate were based on the characteristics of the current headache attack. Despite years of experience, it was often perceived as difficult to determine the severity of an oncoming attack and the need to medicate. During periods in life with increased headache frequency, they viewed themselves as forced to increase their medication use. There was variation in the participants’ awareness and acknowledgement of the link between the increasing frequency of headaches and the use of acute medication.

#### Taking medication because one has to, not because one chooses to

The participants pointed out that they did not like to medicate but felt they had to do so, to manage their headaches. They reported that they strived to use as little medication as possible. Because of this, they did not consider themselves as addicts and they were offended when others, e.g. health-care staff, made them feel like they were.

"It is not really that I am dependent on the medication itself, it is just that I do not want to feel like this. I want to get rid of the headaches and eventually one gets a little bit desperate. (No. 9)"

One reason why they did not want to take medication was concerns about long-term effects. They worried about what would happen to their bodies when they used so much medication for such extended periods e.g. fear that it accumulated in the body somehow.

"Sometimes I worry about taking so many pills. I think, can my liver handle it? I heard that the liver could handle almost anything. You know, thoughts like that. In addition, I get annoyed and worry about side effects but when I have a headache, I forget everything else. I do not care; I would rather die, than to have it this way, because I die every day. That’s how it feels. (No. 6)"

#### Focusing on the headache when deciding whether to medicate

They focused on their headaches and regarded the medication use as a consequence thereof. If headaches started coming more often, the medication use would increase as a result. Decisions about when to medicate were based on the characteristics of the oncoming headache attack. They had tried to endure occasional attacks without medication but this was described as an awful experience that they did not want to repeat. They tried to determine whether the attack was a migraine or not and how severe it would be. Despite years of experience, it was often difficult to determine when to medicate.

"I try to use, in a way, my own experience. What it’s been like previously, what was it like 2 weeks ago and what did I do then? Did I take a tablet or what did I do? (No. 3)"

The concern that the tablets would only be effective if taken early, before the attack had progressed too far, complicated the decision. It stressed them and made them feel that they must decide how to treat the attack before they had had a chance to determine how bad it would become. This complicated the intention to use as little medication as possible.

#### Avoidance of tracking medication use

They were reluctant to think about how much medication they actually used and avoided acquiring a clear overview of their medication use. Many found it difficult to specify how much they used. When asked, they tended to report the number of headache days instead of the number of days with medication use.

"Sometimes almost a week can pass without migraine and then the week after, I have 10 attacks. So it is… a lot of medication. (No. 1)"

Some deliberately avoided keeping track of their medication use and others said that they did not realize the extent of it until they started writing it down and saw the figures. When not keeping a record, they tended to forget and believe they used less than they actually did.

"I try to live in some kind of unawareness of how much [I medicate], and at the same time, I keep thinking I want to take as little medication as possible (No. 5)

Because when you start taking pills, if you don’t write it down very carefully in your diary, you haven’t got the faintest idea! (No. 2)"

#### Increased medication use during stressful periods in life

The headaches were present throughout life and increased during stressful periods, such as after becoming a parent, when changing work place, when moving, during divorce, during unemployment, or in connection with other injuries or illnesses. These were periods of high pressure, and the headaches added an extra burden by becoming even worse than usual. When the frequency of headaches increased, they felt compelled to use more and more medication, particularly if there was no room in life for headaches at that point. They took medication to get rid of the headaches so that they could handle the current situation.

"Then there was a period when I had headaches every day again, constantly, and then I took tablets all the time, every day, and I did not think about it then, I just wanted something that would make it go away… (No. 9)"

#### Perceptions about the link between increasing headaches and medication use

The participants focused on their increasing headache frequency and often did not keep track of their medication use. In their view, they used more and more medication because they had more headaches, not vice versa. They had other explanations as to why the headaches were increasing, e.g. stress, changing hormones or other ailments.

"It feels like the older I get, the more it increases. That is why I have been thinking that it is due to hormones. (No. 7)"

Some had considered the idea that they had MOH, but rejected it since they had not experienced withdrawal symptoms when not taking the medication on occasional days or after making inadequate withdrawal attempts without experiencing improvement.

"Now I don’t have that headache that is caused by the medication, because even if I have migraine attacks… well, say about four times a week, or maybe… well, I don’t take two tablets each time. I take maybe one tablet and then I try to sleep… or I skip it all together, the medication. But it depends on how far it has gone and how much I feel it. (No. 9)"

Others were starting to suspect or realize that the medication use may be causing headache.

"And I think… it started with the headaches but now maybe it is a headache because of the Treo. I do not know. (No. 11)"

## Discussion

This qualitative study generated a substantive theory about the development of MOH. The basic process leading to medication overuse was *holding on to the indispensable medication*. The acute medication was indispensable to the participants because they perceived it as the only thing that could prevent headaches from ruining their lives.

The perception of headaches as a threat to quality of life is consistent with previous research. Quantitative studies have shown reduced quality of life in MOH [3-5], and other qualitative studies have described similar patterns of disability in relation to work, family and social life to those found in this study [27,36,37]. The participants were not passive in relation to this threat. They struggled to uphold their preferred lifestyles despite headaches and invested substantial resources into finding strategies to deal with them. Being actively involved in the management of headache has been reported previously [10]. Peters et al. [10] described active involvement through both decision-making and behaviours. In a study of functional coping, Lauwerier et al. [8] found that those who primarily focused on pain as a problem to solve were at higher risk of developing MOH than those who tried to disengage and focus on other areas of life. The participants in our study also focused on their headaches and invested a lot of effort searching for ways to manage them. In a recent paper, Lauwerier et al. [38] suggested that efforts to control pain may be regarded as attempts to protect valued life goals that are threatened by pain. This could explain why some engage excessively in pain control strategies, such as medication overuse, despite the costs associated with this, such as the development of MOH.

Choosing acute medication as the main strategy to master headache could be regarded as choosing an easy alternative. It requires less effort than many other strategies, e.g. lifestyle changes and therapy. However, the results of this study show that use of acute medication was not the participants’ first choice. They had put a lot of effort into trying to find other strategies. The range of strategies used was similar to that found in a previous qualitative study [25]. Some of the treatments tried lacked scientific evidence but they had also tested treatments that are recommended in official guidelines, such as prophylactic medication and psychotherapy [39,40], without experiencing improvement. In this study, we did not go into the clinical reasons as to why these strategies had not been effective. The participants perceived them as ineffective and consequently, they eventually became resigned, accepting acute medication as the only effective aid. Their reliance on acute medication was thus not a convenient quick solution to the problem; it was rather the only remaining alternative after having tried everything else.

The participants were sceptical about prophylactic medication because they did not like the idea of having to medicate daily. A reluctance to use daily medication has been observed in previous research, e.g. among asthma patients [41]. In the case of MOH, this notion is particularly interesting since persons with MOH are already using acute medication more or less daily [42]. When asked about this, the participants explained that the acute medication was indispensable. Since they were already using so much of this medicine, they felt it was not a good idea to add yet another medication, i.e. the prophylactic. This implies that they somehow viewed the prophylactic and the acute medication as the same thing, i.e. a medication that was harmful and ought to be used as little as possible. By regarding it that way, it is not surprising that they held on to the acute medication rather than the prophylactic. The acute medication had a more obvious effect and only had to be taken when needed. This is an example of how the perspectives of the individual medication user can differ from the traditional medical view. The first step towards successful use of headache medication is probably to bridge the gap between the perspectives of patients and health-care staff.

Lack of information may partly explain why some did not regard their medication use as a causative factor behind the increasing frequency of headaches. A few had never heard of MOH and this implies that there is an unmet need for information concerning this disorder among those at risk of developing it. Further, since we did not try to detoxify the participants, we cannot rule out the possibility that the chronic daily headache of some was indeed caused by factors other than medication overuse. However, despite these possibilities we found it noteworthy that participants who were aware of MOH did not necessarily view the medication as a cause of their own increasing headache frequency. This is somewhat surprising, considering the effort they reported having made in searching for explanations and strategies to treat their headaches. A few participants talked about the association, but our data did not explain why some were aware of it whereas others were not. Realising that medication overuse may be contributing to increased headache seems important for the successful treatment of MOH and thus more research on this stage is needed.

There is an on-going discussion as to whether MOH should be considered an addictive disorder or not [19-23]. The participants in this study expressed that they did not view themselves as addicts and that they felt offended if someone suggested that they were. However, as addiction is sometimes associated with denial, it is difficult to draw conclusions about addiction from this study. An important difference between those with MOH and those with addiction seems to be the reason for the overuse. Addiction is often characterized by a progressive neglect of alternative pleasures or interests because of drug use and may result in a reduction of social, occupational, and recreational activities [8,21]. This is usually not the case in MOH. Instead, both this study and previous research suggest that persons with MOH are overusing the medication to live their lives as normally as possible and reduce the impact of their disorder on their daily lives [8,21]. The participants held on to the medication to prevent the headaches from ruining their lives, not because they wanted the medication per se.

Several studies concerning decision-making among persons with headache suggest that they actively weigh the pros and cons of taking the medication before deciding whether to medicate [9-11]. This corresponds with the beliefs-about-medicines model, concerning chronic illness in general [12]. It hypothesizes that patients engage in an implicit risk-benefit analysis in which beliefs about the necessity of a medication are weighed against concerns about its potential risks. In the case of headache, taking the acute medication is beneficial because the attack is aborted but it also leads to risks in terms of the potential development of MOH. If applying the model strictly, one would expect the risk-benefit analysis to lead to decreased medication use when such negative effects prevail. However, this is not the case in MOH, where many seem inclined to overuse despite being aware of the negative consequences [8,42]. Even after successful withdrawal treatment, often consisting of thorough patient education, the relapse rate is around 30% [6,43]. The model presented in this study provides possible explanations for this behaviour. The perception that headaches are threatening to ruin one’s life and that there are no available solutions other than the acute medication could tip the balance so that the benefits of taking acute medication outweigh the risks. Further, the fact that the participants did not necessarily keep track of their medication use nor think about it as something that contributed to increased headache, probably made it more difficult to conduct the clear-sighted kind of risk-benefit analysis, described by the decision-making models [9-12].

### Methodological considerations

The participants had varied experience and insight into the phenomenon of MOH. Some did not know that MOH existed, others knew about it but did not think that it was the underlying cause of their own increasingly frequent headaches, and some acknowledged that their headache was indeed MOH. Despite this variation, the theoretical pattern relating to the core category applied to all participants. The variation added richness to the theory.

A limitation of the study is that all participants were recruited via advertisements and that we thus only recruited persons who had taken the initiative to talk about their situation. This may e.g. have led to a selection of MOH sufferers who were active and open and thus reinforced the impression that persons with MOH are actively searching for new treatments and new information about their disorder. Interviewing other persons with MOH may possibly have given another picture of the problem. The proportion of participants with a university education was higher than in the general population. However, the external validity in qualitative studies focuses of transferability rather than generalization [44,45] and even though some examples in the data are specific to the participants’ context, they generally expressed the importance of the medication for preventing their disorder from disrupting their lives. This finding may be transferred to persons using headache medication in other settings and even to persons using medications for other disorders.

Analysing the data with another method, such as phenomenology, content analysis or narrative research, would most likely have given the results a different shape as the methods have different theoretical underpinnings and pose different types of questions. In this study, grounded theory was considered the most suitable method, as we wanted to analyse a process. A potential risk with grounded theory is that researchers may allow a preconceived theory to direct the sampling of data and the analysis, thus seeking to verify a preconceived theory rather than finding a new one. However, the structured procedures for data collection and analysis, including the constant comparisons and the asking of questions, are there to prevent such bias [33]. In fact, the methodology emphasises that the theory should be grounded in the data and forces the researcher to constantly redefine the emerging theory as new data is included. In this study, the final theory was very different from the first embryos of the theory that were produced early on during the research process. The multidisciplinary group of co-authors had constant discussions throughout the analysis process in order to prevent preconceptions from affecting the developing theory. Further, the emerging theory was discussed in a multi-disciplinary research seminar and the regular peer scrutiny applied (by TH and AJ) throughout the analysis also adds to the credibility [46]. With these procedures we have done our best to prevent the influence of preconceptions but, as in all qualitative research, the risk can never be entirely eliminated. The model is new and unique, but the essences of several categories are supported by other studies, and this strengthens the credibility of the findings [35].

## Conclusions

The participants in this qualitative study perceived headaches as something that threatened to ruin their lives. As a result, they went to great lengths trying to find strategies to manage their headaches. However, the only strategy actually perceived as effective was the use of acute medication and they eventually became resigned, accepting this as the only effective aid. The acute medication thus became indispensable. They did not like to think about their medication use and avoided keeping track of the amount used. They had a general intention to use as little medication as possible but found themselves compelled to medicate frequently to cope with their headache.

The knowledge gained in this study about the development of MOH from the perspective of the individual with headache may help bridge the gap between different perspectives of medication use. It has the potential to increase understanding between patients and health-care professionals and may thereby contribute to improved care.

## Competing interests

One of the co-authors, Mattias Linde is the member of an Allergan International advisory board and receives honoraria in connection with that work. Apart from that, we report no financial or other relationships that may lead to a conflict of interest.

## Authors’ contributions

PJ was involved in the planning of the study, managed the data collection, did the interviews, took the main responsibility for the analysis and drafted the manuscript. AJ was involved in the planning of the study, was actively involved in the analysis of the data, the development of the categories, the interpretation of the results and contributed to the writing of the manuscript. GH was involved in the planning of the study, the development of the categories and the interpretation of the results, and contributed to the writing of the manuscript. ML was involved in the planning of the study, the data collection, participated in the interpretation of the results, and contributed to the writing of the manuscript. CDM was involved in the development of the categories and the interpretation of the results, and contributed to the writing of the manuscript. TH was involved in the planning of the study, was actively involved in the analysis of the data, the development of the categories, the interpretation of the results and contributed to the writing of the manuscript. All authors read and approved the final manuscript.

## References

[B1] Olesen J Bousser MG Diener HC Dodick D First M Goadsby PJ Gobel H Lainez MJ Lance JW Lipton RB Nappi G Sakai F Schoenen J Silberstein SD Steiner TJ New appendix criteria open for a broader concept of chronic migraine Cephalalgia 2006 14 6 742 746 16686915 10.1111/j.1468-2982.2006.01172.x

[B2] Katsarava Z Schneeweiss S Kurth T Kroener U Fritsche G Eikermann A Diener HC Limmroth V Incidence and predictors for chronicity of headache in patients with episodic migraine Neurology 2004 14 5 788 790 10.1212/01.WNL.0000113747.18760.D2 15007133

[B3] D“Amico D Grazzi L Usai S Rigamonti A Curone M Bussone G Disability pattern in chronic migraine with medication overuse: a comparison with migraine without aura Headache 2005 14 5 553 560 10.1111/j.1526-4610.2005.05109.x 15953274

[B4] Wiendels NJ Knuistingh Neven A Rosendaal FR Spinhoven P Zitman FG Assendelft WJ Ferrari MD Chronic frequent headache in the general population: prevalence and associated factors Cephalalgia 2006 14 12 1434 1442 10.1111/j.1468-2982.2006.01210.x 17116093

[B5] Colas R Munoz P Temprano R Gomez C Pascual J Chronic daily headache with analgesic overuse: epidemiology and impact on quality of life Neurology 2004 14 8 1338 1342 10.1212/01.WNL.0000120545.45443.93 15111671

[B6] Evers S Marziniak M Clinical features, pathophysiology, and treatment of medication-overuse headache Lancet Neurol 2010 14 4 391 401 10.1016/S1474-4422(10)70008-9 20298963

[B7] Craig DG Bates CM Davidson JS Martin KG Hayes PC Simpson KJ Staggered overdose pattern and delay to hospital presentation are associated with adverse outcomes following paracetamol-induced hepatotoxicity British journal of clinical pharmacology 2012 14 2 285 294 10.1111/j.1365-2125.2011.04067.x 22106945 PMC3269587

[B8] Lauwerier E Paemeleire K Van Damme S Goubert L Crombez G Medication use in patients with migraine and medication-overuse headache: the role of problem-solving and attitudes about pain medication Pain 2011 14 6 1334 1339 10.1016/j.pain.2011.02.014 21396772

[B9] Ivers H McGrath PJ Purdy RA Hennigar AW Campbell MA Decision making in migraine patients taking sumatriptan: an exploratory study Headache 2000 14 2 129 136 10.1046/j.1526-4610.2000.00018.x 10759912

[B10] Peters M Abu-Saad HH Vydelingum V Dowson A Murphy M Patients“ decision-making for migraine and chronic daily headache management A qualitative study. Cephalalgia 2003 14 8 833 841 10.1046/j.1468-2982.2003.00590.x 14510931

[B11] Katic BJ Krause SJ Tepper SJ Hu HX Bigal ME Adherence to acute migraine medication: what does it mean, why does it matter? Headache 2010 14 1 117 129 10.1111/j.1526-4610.2009.01535.x 19817884

[B12] Horne R Weinman J Patients“ beliefs about prescribed medicines and their role in adherence to treatment in chronic physical illness Journal of psychosomatic research 1999 14 6 555 567 10.1016/S0022-3999(99)00057-4 10661603

[B13] Byrne M Walsh J Murphy AW Secondary prevention of coronary heart disease: patient beliefs and health-related behaviour Journal of psychosomatic research 2005 14 5 403 415 10.1016/j.jpsychores.2004.11.010 16026655

[B14] Menckeberg TT Bouvy ML Bracke M Kaptein AA Leufkens HG Raaijmakers JA Horne R Beliefs about medicines predict refill adherence to inhaled corticosteroids Journal of psychosomatic research 2008 14 1 47 54 10.1016/j.jpsychores.2007.07.016 18157999

[B15] Horne R Weinman J Self-regulation and self-management in astma: exploring the role of illness perceptions and treatment beliefs in explaining non-adherence to preventive medication Psychol Health 2002 14 17 10.1080/08870440290001502

[B16] Horne R Weinman J The beliefs about medicines questionnaire: The development and evaluation of a new method for assessing the cognitive representation of medication Psychol Health 1999 14 1 10.1080/08870449908407311

[B17] Phatak HM Thomas J 3rd Relationships between beliefs about medications and nonadherence to prescribed chronic medications Ann Pharmacother 2006 14 10 1737 1742 10.1345/aph.1H153 16985088

[B18] Mardby AC Akerlind I Jorgensen T Beliefs about medicines and self-reported adherence among pharmacy clients Patient education and counseling 2007 14 1–3 158 164 17913439 10.1016/j.pec.2007.08.011

[B19] Sances G Galli F Anastasi S Ghiotto N De Giorgio G Guidetti V Firenze C Pazzi S Quartesan R Gallucci M Nappi G Medication-overuse headache and personality: a controlled study by means of the MMPI-2 Headache 2010 14 2 198 209 10.1111/j.1526-4610.2009.01593.x 20039955

[B20] Radat F Lanteri-Minet M What is the role of dependence-related behavior in medication-overuse headache? Headache 2010 14 10 1597 1611 10.1111/j.1526-4610.2010.01755.x 20807250

[B21] Ferrari A Cicero AF Bertolini A Leone S Pasciullo G Sternieri E Need for analgesics/drugs of abuse: a comparison between headache patients and addicts by the Leeds Dependence Questionnaire (LDQ) Cephalalgia 2006 14 2 187 193 10.1111/j.1468-2982.2005.01020.x 16426274

[B22] Fuh JL Wang SJ Lu SR Juang KD Does medication overuse headache represent a behavior of dependence? Pain 2005 14 1–3 49 55 16298069 10.1016/j.pain.2005.09.034

[B23] Calabresi P Cupini LM Medication-overuse headache: similarities with drug addiction Trends Pharmacol Sci 2005 14 2 62 68 10.1016/j.tips.2004.12.008 15681022

[B24] Radat F Creac“h C Guegan-Massardier E Mick G Guy N Fabre N Giraud P Nachit-Ouinekh F Lanteri-Minet M Behavioral dependence in patients with medication overuse headache: a cross-sectional study in consulting patients using the DSM-IV criteria Headache 2008 14 7 1026 1036 10.1111/j.1526-4610.2007.00999.x 18081820

[B25] Peters M Abu-Saad HH Vydelingum V Dowson A Murphy M Migraine and chronic daily headache management: a qualitative study of patients“ perceptions Scand J Caring Sci 2004 14 3 294 303 10.1111/j.1471-6712.2004.00279.x 15355524

[B26] Hansen DL Hansen EH Holstein BE Using analgesics as tools: young women“s treatment for headache Qual Health Res 2008 14 2 234 243 10.1177/1049732307312303 18216342

[B27] Leiper DA Elliott AM Hannaford PC Experiences and perceptions of people with headache: a qualitative study BMC Fam Pract 2006 14 27 10.1186/1471-2296-7-27 16670013 PMC1523257

[B28] Axelsson M Lotvall J Lundgren J Brink E Motivational foci and asthma medication tactics directed towards a functional day BMC public health 2011 14 809 10.1186/1471-2458-11-809 21999635 PMC3210153

[B29] Sanderson T Morris M Calnan M Richards P Hewlett S What outcomes from pharmacologic treatments are important to people with rheumatoid arthritis? Creating the basis of a patient core set Arthritis care & research 2010 14 5 640 646 10.1002/acr.20034 20461785 PMC2887082

[B30] Green J Britten N Qualitative research and evidence based medicine Bmj 1998 14 7139 1230 1232 10.1136/bmj.316.7139.1230 9583929 PMC1112988

[B31] Peters M Abu-Saad HH Vydelingum V Murphy M Research into headache: the contribution of qualitative methods Headache 2002 14 10 1051 1059 10.1046/j.1526-4610.2002.02238.x 12453040

[B32] Linde M Gustavsson A Stovner LJ Steiner TJ Barre J Katsarava Z Lainez JM Lampl C Lanteri-Minet M Rastenyte D Ruiz de la Torre E Tassorelli C Andree C The cost of headache disorders in Europe: the Eurolight project Eur J Neurol 2011 10.1111/j.1468-1331.2011.03612.x 22136117

[B33] Corbin J Strauss A Basics of qualitative research: techniques and procedures for developing grounded theory 2008 3 Thousand Oaks: SAGE

[B34] Schreiber RS Stern PN ebrary Inc Using grounded theory in nursing 2001

[B35] Dellve L Henning-Abrahamsson K Trulsson U Hallberg L Hallberg LRM Grounded theory in public health research Qualitative methods in public health research : theoretical foundations and practical examples 2002 Lund: Studentlitteratur 137 175

[B36] Peters M Huijer Abu-Saad H Vydelingum V Dowson A Murphy M The patients“ perceptions of migraine and chronic daily headache: a qualitative study J Headache Pain 2005 14 1 40 47 10.1007/s10194-005-0144-7 16362190 PMC3451956

[B37] Tenhunen K Elander J A qualitative analysis of psychological processes mediating quality of life impairments in chronic daily headache J Health Psychol 2005 14 3 397 407 10.1177/1359105305051425 15857870

[B38] Lauwerier E Van Damme S Goubert L Paemeleire K Devulder J Crombez G To control or not? A motivational perspective on coping with pain Acta neurologica Belgica 2012 14 1 3 7 10.1007/s13760-012-0020-6 22427282

[B39] Bendtsen L Evers S Linde M Mitsikostas DD Sandrini G Schoenen J EFNS guideline on the treatment of tension-type headache - report of an EFNS task force Eur J Neurol 2010 14 11 1318 1325 20482606 10.1111/j.1468-1331.2010.03070.x

[B40] Evers S Afra J Frese A Goadsby PJ Linde M May A Sandor PS EFNS guideline on the drug treatment of migraine–revised report of an EFNS task force Eur J Neurol 2009 14 9 968 981 10.1111/j.1468-1331.2009.02748.x 19708964

[B41] Adams S Pill R Jones A Medication, chronic illness and identity: the perspective of people with asthma Soc Sci Med 1997 14 2 189 201 10.1016/S0277-9536(96)00333-4 9225407

[B42] Jonsson P Linde M Hensing G Hedenrud T Sociodemographic differences in medication use, health-care contacts and sickness absence among individuals with medication-overuse headache J Headache Pain 2012 14 4 281 290 10.1007/s10194-012-0432-y 22427000 PMC3356474

[B43] Katsarava Z Muessig M Dzagnidze A Fritsche G Diener HC Limmroth V Medication overuse headache: rates and predictors for relapse in a 4-year prospective study Cephalalgia 2005 14 1 12 15 10.1111/j.1468-2982.2004.00789.x 15606564

[B44] Malterud K Qualitative research: standards, challenges, and guidelines Lancet 2001 14 9280 483 488 10.1016/S0140-6736(01)05627-6 11513933

[B45] Whittemore R Chase SK Mandle CL Validity in qualitative research Qual Health Res 2001 14 4 522 537 10.1177/104973201129119299 11521609

[B46] Shenton A Strategies for ensuring trustworthiness in qualitative research projects Education for Information 2004 14 63 75

